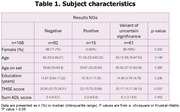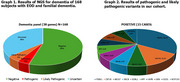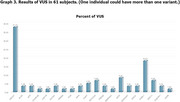# Early Onset Dementia: Experience from Thailand

**DOI:** 10.1002/alz70855_102587

**Published:** 2025-12-23

**Authors:** Vorapun Senanarong, Chatchawan Rattanabannakit, Natthamon Wongkom, Pathitta Dujada, Atthapon Raksthaput, Sunisa Chaichanettee, Theeraphong ‐ Pho‐iam, Chanin Limwongse, Philip Scheltens, Pedro Rosa‐Neto, Serge Gauthier

**Affiliations:** ^1^ Faculty of Medicine Siriraj Hospital, Mahidol University, Bangkoknoi, Bangkok, Thailand; ^2^ Faculty of Medicine Siriraj Hospital, Mahidol University, Bangkok, Thailand; ^3^ Department of Medicine, Faculty of Medicine Siriraj Hospital, Mahidol University, Bangkok, Bangkok, Thailand; ^4^ Division of Neurology, Department of Medicine, Faculty of Medicine Siriraj Hospital, Mahidol University, Bangkok, Thailand; ^5^ Siriraj Genomics, Office of the Dean, Faculty of Medicine Siriraj Hospital, Mahidol University, Bangkok, Thailand, Bangkok, Thailand; ^6^ Division of Medical Genetics, Department of Medicine, Faculty of Medicine Siriraj Hospital, Mahidol University, Bangkok, Thailand, Bangkok, Thailand; ^7^ EQT Life Sciences Partners, Amsterdam, 1071 DV Amsterdam, Netherlands; ^8^ Alzheimer Center Amsterdam, Neurology, Vrije Universiteit Amsterdam, Amsterdam UMC location VUmc, Amsterdam, Netherlands; ^9^ McGill University Research Centre for Studies in Aging, Montreal, QC, Canada; ^10^ McConnell Brain Imaging Centre, Montreal Neurological Institute, McGill University, Montreal, QC, Canada; ^11^ Department of Neurology and Neurosurgery, and Department of Psychiatry, McGill Centre for Studies in Aging, McGill University, Montreal, QC, Canada; ^12^ McGill University, Montreal, QC, Canada

## Abstract

**Background:**

Early‐onset dementia (EOD) is a uncommon form of dementia that afflicts people before age 65. Only a few studies analyzing the genetics of EOD have been performed in Thai population. EOD remains a challenge due to the diverse genetic and clinical heterogeneity of these diseases. The aim of this study was to investigate the genetic spectrum of Thai EOD and those with familial history of dementia.

**Method:**

150 subjects with EOD AND 18 individuals with familial dementia were recruited. Targeted next generation (NGS) analyses were performed to screen 38 genes associated with dementia.

**Result:**

Subject characteristics were demonstrated in table 1. Fifteen had pathogenic variants. Among pathogenic (47%) and likely pathogenic (53%) variants, 5 (33.33%) were in *PSEN1* (c.417G>T, p.Met139Ile), (c.344A>G, p.Tyr115Cys), (c.817G>A, p.Glu273Lys), (c.817G>A, p.Glu273Lys), (c.485T>C, p.Ile162Thr)); 2 (13.33%) in *CSF1R* (c.704T>G, p.Val235Gly), (c.2522A>G, p.Tyr841Cys); 1(6.67%) each in *ABCA7* (c.5571‐1G>C, p.?), *SNCB* (c.372G>A, p.Gln124=), *SORL1* (c.2212G>A, p.Gly738Arg), *APP* (c.2149G>A, p.Val717Ile), *VAPB* (c.301G>T, p.Asp101Tyr), *GRN* (c.276C>A, p.Cys92Ter), *SOD1* (c.143T>C, p.Val48Ala), and *NOTCH3* (c.1630C>T, p.Arg544Cys). Among these, the *PSEN1* variant c.817G>A (p.Glu273Lys) had not previously been reported. 61 had variants of uncertain significance (VUS). Graphs 1‐3 showed results of NGS for dementia in the whole cohort, in those with pathogenic variants, and in those with VUS.

**Conclusion:**

Our study demonstrated the genetic spectrum of EOD and familial dementia in Thai patients. The utilization of next‐generation sequencing could help deciphering the genetic causes of Alzheimer's disease. The genetic testing of known causal genes in EOD patients can help make a precise diagnosis.

We acknowledged Thailand Science Research and Innovation for supporting this study.